# How do we respond to the threat of multidrug-resistant bacteria? Comparison of antibiotic appraisals from 2016 to 2020 of the French, English, and German HTA agencies

**DOI:** 10.1017/S0266462324000552

**Published:** 2024-12-09

**Authors:** Rémy Dumont, Etienne Lengliné, Clara Delorme, Jean-Pierre Bru, Sévérine Ansart, Elisabeth Aslangul, Sophie Kelley, Pierre Cochat, Sylvie Chevret, Thierno Diatta

**Affiliations:** 1 Assessment and Access to Innovation Direction, Haute Autorité de Santé, Saint-Denis, France; 2 Haematology Department, AP-HP Saint Louis Hospital, Paris, France; 3 Infectious Diseases, Annecy Genevois Hospital, CHANGE, Annecy, France; 4 Department of Infectious Diseases, University Hospital of Brest, Brest, France; 5 Service de Médecine Interne, Hôpital Louis-Mourier, Assistance Publique-Hôpitaux de Paris, Colombes, France; 6 UPD5, Université Paris-Descartes, Paris, France; 7 Scientific Board, Haute Autorité de Santé, Saint-Denis, France; 8 ECSTRRA Team, Université Paris Cité, Paris, France

**Keywords:** antimicrobial resistance, health technology assessment, antibiotics, multidrug-resistant bacteria

## Abstract

Antimicrobial resistance (AMR) has become a worldwide growing concern over the past decades. Thus, encouraging manufacturers to develop new antibiotics is needed. We hypothesised that transparency on the regulatory appraisals of antibiotics would provide an incentive to pharmaceutical development. We thus aimed at reporting the French health technology assessment (HTA) opinions and reimbursement decision on antibiotics to those German (G-BA) and English (NICE) HTA bodies.

A qualitative analysis of the Transparency Committee of the French National Authority for Health (TC-HAS) opinions regarding antibiotics assessment between 2016 and 2020 was performed. Decisions of reimbursement by TC-HAS were compared to those from G-BA and NICE when available. TC-HAS recognized a clinical benefit (CB) for 15/15 evaluated indications, a clinical added value for 9/15, and a public health interest for 8/15. Among the valued antibiotics by HAS, 5 were recommended for restricted use as a “reserve” to protect against the risk of resistance emergence. A comparison of HTA opinions was possible across HTA for only 8 antibiotics. The G-BA granted a reserve status for 4 drugs and NICE a reserve with restricted use for 5 antibiotics. Three of these antibiotics were positioned similarly by the English, German, and French HTA bodies. This qualitative analysis of HTA opinions between different European HTA bodies shows a consistent reimbursement decision of antibiotics against MDR bacteria and tuberculosis besides the differences in the applied assessment methods. This work also shows how HTA bodies could recognize a clinical added value in a context of the emergence of antibiotic resistance.

## Introduction

Antimicrobial resistance (AMR) has become a growing concern worldwide in the 21st century, with multifaceted drivers that include overuse and inappropriate use of antibiotics. The ease of global travel has accelerated the spread of resistant bacteria, and antibiotic resistance is becoming a critical global health threat. Indeed, 1.27 million global deaths were attributed to bacterial AMR in 2019, projected to 10 million by 2050 if drug-resistant infections persist (1,2).

In 2016, the World Health Organization (WHO) established a priority list of antibiotic-resistant bacteria to guide research and development (3). The WHO coordinating group listed 20 bacterial species with 25 patterns of acquired resistance and 10 criteria to assess priority. The critical-priority bacteria included carbapenem-resistant *Acinetobacter baumannii* and *Pseudomonas aeruginosa*, and carbapenem-resistant and third-generation cephalosporin-resistant *Enterobacteriaceae* (Table S1). They concluded that future development strategies should focus on antibiotics active against multidrug-resistant (MDR) tuberculosis and Gram-negative bacteria (GNB) given their large public health implications.

To limit resistance development and effectively treat infections, principles of antimicrobial use involve prescription, education, surveillance, and infection control. In antibiotics appraisal, all health technology assessment (HTA) bodies have moreover introduced the concept of “reserve” antibiotics, designating antibiotics as last-resort options when all the others have failed. However, according to the 2022 WHO pipeline study, only 54 percent of developed antibiotics target such pathogens, and only 4 had new modes of action (4). This emphasizes the need for investment to address unmet patient needs (5).

However, the conventional business model for pharmaceutical companies is challenging. Indeed, investing in new antimicrobials may appear not commercially attractive because of their targeted action, limited spectrum, and risk of resistance, faced to strict controls restricting their use (6,7). Thus, several countries have explored ‘pull incentives’ to encourage investing in the research and development of new antibiotics (8,9). We focused on the regulatory and economic incentives provided by the HTA of three countries (10–12), each sharing about 20 percent of the pharmaceutical European drugs market, namely Germany (21 percent), United Kingdom (20 percent), and France (18 percent) – the European market representing 23.4 percent of global market share (13).

### French HTA body (HAS) and actions on AMR

In France, the Transparency Committee of the French National Authority for Health (TC-HAS) provides an independent appraisal, based on 3 measures of any medicinal product submitted by the firm to reimbursement by the National Health Insurance (NHI) Fund, with published guidance (14–16). First, the CB (ranked insufficient, weak, moderate, or important) is based on its efficacy/adverse effects ratio, place in the therapeutic strategy, seriousness of the disease, preventive/curative/symptomatic aim, and public health impact (PHI); it impacts its reimbursement by the NHI. Second, the clinical added value (CAV) compares the efficacy and safety of the product with that of existing alternatives, ranked on a 5-level class from major (level I) down to none (level V). It is used by the French Healthcare Products Pricing Committee (CEPS) to negotiate prices with manufacturers. Third, the PHI indicates the product benefit compared to that of the alternatives in terms of public health (improving the population health, addressing unmet medical needs, or reducing resource consumption).

For reserve antibiotics, a specific section has been addressed in the explicitly published TC-HAS guidance aiming at enlightening the manufacturers on its expectations regarding the CAV of the product, stressing out the importance to address unmet medical needs (notably with regards to MDR strains) (14).

### German HTA body (G-BA) and actions on AMR

In Germany, the Federal Joint Committee (G-BA) ranks the CAV of the product over that of a clinically appropriate comparator, based on a 6-class scale (from major to lesser benefit). Once one year in the market, the drug price is negotiated with social insurances. Then, final assessments and decisions of G-BA are published online (17).

With the support of the Federal Institute for Medicines (BfArM), the Robert Koch Institute (RKI) published in February 2021 a selection of pathogens involved in MDR bacterial infections as a national basis (Table S2), derived from the WHO list of priority bacteria (3). It proposed criteria for the classification of a newly authorized antibiotic as a reserve antibiotic (18) (Table 1). For these reserve antibiotics, the “German Act on Fair Competition between Health Insurance Funds in Statutory Health Insurance,” has been implemented to exempt those antibiotics from full HTA, providing substantiated additional CB without appropriate studies (19). Nevertheless, if the antibiotic is prescribed “much more frequently than expected from a prescription at strictly regulated indications” or in case of “large scale” off-label use, a full assessment is required.

**Table 1.** German Robert Koch Institute (RKI) indicator list for classifying a new antibiotic as a reserve antibiotic

Criterion	Definition	Indicator
Efficacy against relevant multidrug-resistant pathogens	1.* The NAB is effective against at least one pathogen in accordance with the classification on the Pathogen List adapted for Germany^a^	1.1 The NAB has a marketing authorization for a pathogen-specific indication (as per EMA/844951/2018 Rev. 3) for the treatment of infections with MRP (in accordance with Pathogen List classification) in patients with limited therapeutic options.^b^
		1. The NAB does not have a marketing authorization in accordance with 1.1 but does have a marketing authorization for the treatment of at least one specific, potentially serious infection, and efficacy against MRP (in accordance with Pathogen List classification) has been shown. 1.2.1* Meaningful data substantiate the in vitro efficacy against a relevant MDR pathogen. **And** 1.2.2 Results of at least one clinical study show clinical efficacy against this relevant MDR pathogen (≥ 10 patients).
Treatment especially of serious bacterial infections without or with limited clinically equivalent treatment options	2. The NAB is the only or one of few treatment options for the targeted therapy of infections with relevant pathogens (see Pathogen List^a^) or the prophylaxis of appropriate serious disorders (within the authorized indications) (securing of treatability).	2.1* Guideline review: For the authorized indication(s) in connection with relevant MRP (in accordance with Pathogen List^a^), no or only limited clinically equivalent therapeutic options or possibilities of prophylaxis are available (with reference to the entire or to a specific patient cohort, e.g., children). (2.1 is already confirmed with a marketing authorisation in accordance with 1.1.)

NAB, new antibiotic; MRP, multiresistant pathogen.

a See methodology for compiling the Pathogen List as per Section 35a subsection 1c sentence 6 German Social Code, Book V.

b A marketing authorisation for a pathogen-specific indication as per EMA/84451/2018 Rev. 3 for the treatment of infections with multidrug resistant in patients with limited therapeutic options is a criterion in its own right, as reserve status and an unmet medical need has already been confirmed in the regulatory procedure.

**Explanatory notes**

A scientific assessment of the criteria takes place for the antibiotic per authorized indication.

*To 1.:

The Pathogen List concerns an inconclusive list, i.e., in individual cases, the examination of an application for reserve status can also take place in the case of efficacy against other, unlisted MRP, for example, owing to high clinical relevance and a lack of therapeutic options.

*To 1.2.1:

Methodology/evaluation: The clinical isolates for the examination of the in vitro efficacy should originate from relevant, meaningful samples, be representative of Germany, and should have been examined within the last five years. In the case of commonly occurring pathogens, in vitro examination should take place on the strength of several hundred isolates, and in the case of rare pathogens, on the strength of at least 10 isolates. An appropriate justification of the frequency and number of isolates selected should be presented. Measurement of the minimum inhibitory concentration (MIC) as well as assessment applying and accounting for the MIC limits and methodology of European Committee on Antimicrobial Susceptibility Testing (EUCAST) (11) should take place. The MIC of the antibiotic applied should be compared to the in vitro sensitivity of several antibiotics recommended in current guidelines or publications for the authorized indication(s).

Efficacy in vitro:

The in vitro efficacy of an antibiotic against a relevant MDR pathogen can be concluded if the results of the in vitro sensitivity of the clinical isolates are predominantly in the sensitive range of the EUCAST limits, and the proportion of sensitively tested isolates is comparable to or better than recommended alternative antibiotics. The resistance rates to the antibiotics examined should be compared and discussed in the evaluation.

*To 2.1.:

Guideline review:

A guideline review should take place based on current national guidelines with a high evidence level. If no appropriate current national guidelines are available for the authorized indication(s), the review may be based on current European or international guidelines, with an evidence level. Additionally, it can be shown, based on the strength of current literature, that for the authorized indication(s) in connection with relevant MRP (in accordance with the Pathogen List), no or only limited clinically equivalent therapeutic options or possibilities of prophylaxis are available.

To regulate and encourage the proper use of antibiotics, the G-BA also uses financial mechanisms. For instance, in 2023, all reserve antibiotics launched before 2031 have been excluded from the normal pricing negotiation under the drug pricing law AMNOG (unlimited free pricing), with the manufacturer will freely determine the retail price of the drug (20).

### English HTA body (NICE) and actions on AMR

The HTA body in England and Wales, the National Institute for Health and Care Excellence (NICE), proposes reimbursement and determines pricing for selected drugs, considering both clinical and cost-effectiveness to their appraisal. Any drug to be used in England must be listed on the public formulary, decided upon the NICE based on its incremental cost-effectiveness ratio (ICER), if the price of the drug, negotiated with the manufacturer, is compatible with the public acceptability threshold of £20,000 to £30,000 (possibly increased) per quality-adjusted life-year (QALY). Five types of recommendations are possible: recommended, optimized, Cancer Drugs Fund, not recommended, and only in research (21).

Moreover, solutions that evaluate and pay for selected antibiotics in a different way from other medicines have also been tested. The national action plan for AMR, published in January 2019, introduced the “Antimicrobial Products Subscription Model” jointly with the National Health Service (NHS) to provide a guaranteed return on investment for two selected drugs, ceftazidime with avibactam and cefiderocol, after regulatory approval (22,23). The model used a qualitative framework with a points-based scoring system to determine the contract value for the antibiotic, instead of using health economic modeling to estimate QALY.

An academic approach has also been carried out by both the Office of Health Economics and the Academy of Infection Management with the participation of private partners on the elements to consider in the HTA of new antibiotics (24). Value elements were divided into two groups: relevant benefits generally included in HTA (health gain, unmet need, and cost offsets), and other types of benefits that might be relevant for antibiotics (Table 2).

**Table 2.** UK criteria for appraisal of new antibiotics proposed by the Office of Health Economics and the Academy of Infection Management

Element of value	Indicator of value	Evidence of value	Is the indicator of value and evidence of Value typically accepted by HTA bodies?	Issue particularly relevant to antibiotics?
**Relevant benefits typically included in traditional HTA**				
Health gain	Short-term clinical efficacy: cure of infection Long-term clinical efficacy: reduced recurrent infections and reduced long-term mortality	Noninferiority trials	X	✓
		Superiority trials	✓	X
		Non-RCT evidence of superiority, e.g., RWE	X	✓
	Microbiological efficacy	Non-RCT evidence of superiority, e.g., microbiology, PK/PD data	X	✓
	Quality of life improvement (short-term and long-term)	Noninferiority trials	X	X
		Superiority trials	✓	X
	Availability of rapid molecular diagnostic	Evidence of test accuracy	X	✓
Unmet need	Severity of disease	Evidence of length/quality of life with SOC	X	X
		Priority pathogen lists	X	✓
	Availability of alternative treatments, e.g., other effective antibiotics	Epidemiology studies (AMR rates for particular pathogens)	X	✓
Cost offsets	Reduction in use of other treatments/services or length of hospital stays	Modeling studies/clinical trials	✓	X
Productivity benefits	Presenteeism/absenteeism	Modelling studies	X	X
**Other types of benefit of relevance to antibiotics (not included in traditional HTA)**				
Transmission value	Reducing overall incidence of an infection	Epidemiology studies	X (except for vaccine)	✓
Insurance value	Protection from (non) catastrophic event	Modelling studies	X	✓
Diversity value	Reduced AMR due to “rest period”	Modelling studies	X	✓
Novel action value	New mechanism of action	Evidence of new or unique mechanism of action	X	✓
Enablement value	Enablement of treatment, e.g., prophylactic use in surgery or chemotherapy	Modelling studies	X	✓
Spectrum value	Narrow versus broad spectrum	Depends on AB	X	✓

Key: ‘✓’ means that the answer to the questions posed in Columns 4 and 5 is ‘yes’. ‘X’ means that the answer to the questions posed in Columns 4 and 5 is “no.”’

* Note that transmission value is considered in vaccine assessment and decision-making. However, many HTA bodies assessing drugs do not assess vaccines.

### EU actions on AMR

The European Commission has become aware of the threat of AMR in the early 2000s. Strategies (2001), recommendations (2008) and EU-wide plans to combat AMR (2011 and 2017) have marked the Commission’s reflections and actions. In June 2023, the Council adopted the Recommendation on stepping up EU actions to combat AMR in a “One Health” approach. On 13 February 2024, the Commission launched a new project focusing on AMR and healthcare-associated infections. The objective of this project is to reduce the risk of exposure of citizens to antibiotic-resistant bacteria and is supported with €50 million under the EU4Health programme (25).

At the present time, potential financing models are vetted through the European Joint Action on AMR and Healthcare-Associated Infections (EU-JAMRAI): 1/ diagnosis-related group carve-out, allowing hospital antibiotics to be reimbursed at higher prices and potentially removes any economic disincentive for use; 2/ stewardship taxes, for instance applied to human antibiotic consumption, 3/ transferable exclusivity voucher awarded to the innovator of a novel antibiotic meeting predefined specifications that can then be used to extend the monopoly time period of any patented medicine, and 4/ a European-based “pay or play” model on all marketing authorizations (MAs) (human and veterinary) to the European Medicines Agency (EMA), except those for human antibiotics (or alternatives) (26). In this way, all nonantibiotics would pay for antibiotic innovation.

When efforts are made to promote the proper use of antibiotics, an impact on the development of AMR is expected. As an example, the French program against antibiotic resistance has been intensified since 2000. It appears successful in decreasing antibiotic consumption (from 19.9 defined daily doses/1,000 inhabitants per day in 2019 to 16.4 in 2020), and antibiotic resistance for some microorganisms (such as *Escherichia coli* isolates resistant to fluoroquinolones, dropping from 20.0 percent in 2017 down to 18.7 percent in accommodation facilities for dependent elderly people in 2022) (27,28). Moreover, in 2019, French guidelines emerged concerning the utilization of carbapenems and their alternatives for treating *enterobacteria* and *P. aeruginosa* infections in adults. Within the scope of the French program against antibiotic resistance, a retrospective analysis was initiated, for insight into how to align the principles guiding the assessment of these antibiotics with the prevailing guidance of medicinal product evaluation. We hypothesized that the transparency on the regulatory appraisals and reimbursement decisions of new antibiotics by the HTA would provide an incentive to the pharmaceutical development in this area of research. We thus analyzed and detailed the appraisals of new antibiotics assessed by the French TC-HAS from 2016 to 2020, then compared these appraisals to those from the NICE and the G-BA.

## Methods

### Role of the working group

A working group (WG) was composed of volunteered TC-HAS members (microbiologists, physicians, and methodologists) and two HAS supports of the Transparency Commission, responsible for the medical and administrative management of applications for reimbursement purposes and specialist in infectious diseases. First, the WG reviewed all the consecutive TC-HAS opinions on those antibiotics over a 5-year period. Secondly, the WG performed a comparison of TC-HAS decisions to those reported by the NICE and G-BA. Last, the WG presented the results of these comparisons and the EMA guidance on the development of new antibiotics (29), leading the TC-HAS to update its guidance by incorporating the specificities of the assessment of new antibiotics, as detailed below.

### Extraction and analysis of available HTA opinions

The study was scheduled in January 2021, with all the TC-HAS assessment of antibiotics made from January 1, 2016, to December 31, 2020 considered for analysis. The date of onset was chosen given that the first antibiotic pairing a cephalosporin with a beta-lactamase inhibitor was evaluated by the TC-HAS in July, 2016.

The data extraction was carried out on January 13, 2021. Evidence was extracted into a database created in Microsoft Excel for coding and analysis. Information extracted from TC-HAS opinions were the date of assessment, drug class, MA indications, context of the assessment (first inscription, reassessment [new assessment based on new additional clinical data], or additional indication), target population, design, and objectives of pivotal trials (if any), TC-HAS final opinion on CB, PHI and CAV, and specific expert recommendations. This information was analyzed to highlight the essential elements for the valuation of an antibiotic, either in terms of the type of clinical data or the methodology used by the manufacturer, and to highlight which elements the TC-HAS was expecting to remove any uncertainties linked to the assessment of a therapeutic advance. This analysis was made without adjustment and was reported in percentages.

Then, a qualitative comparison of the decisions taken by France to those from England and Germany, using available opinions published on their websites, was conducted. It assessed the similarities and differences between these HTA bodies in terms of the NHI system coverage decisions and scopes of reimbursement, i.e., within the MA indications or with restrictions. The lack of quantitative comparison was dictated by the low sample of assessed antibiotics. Moreover, English and German opinions were not all available to allow a comparison of all submissions due to the specificities and HTA rules of each body, as reported above (30,31).

## Results

### Key insights from the TC-HAS assessments

Between January 1, 2016 and December 31, 2020, TC-HAS performed 15 assessments (6 in 2016, 2 in 2017 and 7 in 2020) on 12 different antibiotics for reimbursement purposes (32–46): they consisted of one third-generation cephalosporin (5 indications), 2 other cephalosporins and penems (7 indications), 1 fluoroquinolone (1 indication), 1 broad-spectrum penicillin (pivmecillinam, 1 indication), 1 glycopeptide (1 indication), 1 tetracycline (2 indications), 1 trimethoprim (1 indication), 2 carbapenems (6 indications), and 2 targeted MDR tuberculosis.

Table 3 shows the main characteristics of data available at the time of assessment, with resulting CB, CAV, and PHI-graded values. Complicated intra-abdominal infection (cIAI) and complicated urinary tract infection (cUTI) treatment accounted for 6 and 5 of the indications, respectively. The remaining indications included various conditions, including acute bacterial skin and skin structure infections (ABSSSI, 5 indications) and ventilator-associated pneumonia (VAP, 4 indications). One drug (delamanide) was granted a pediatric MA based on a single-arm study, while 9 (60 percent) followed a randomized controlled trial (RCT), mostly based on a noninferiority design (n = 8/9); the remaining drugs were used either based on bibliographic data only (n = 3) or only based on pharmacological data (n = 3).

**Table 3.** Appraisal of 12 antibiotics assessed between 2016 and 2020 by French (HAS), English (NICE), and German (G-BA) HTA bodies

Drug					Available data			TC-HAS							NICE		G-BA		
Drug Class	ATC	INN (Brand name)	Indication	Population	Design	Comp.	Obj.	Date	*	Context	CB	CAV	PHI	Status	Date	Status	Date	CAV	Status
antimycobacterial	J04AK06	delamanid	MDR-TB	Adult	Phase II	Placebo	S	jan.–16	*	I	S	III	–				may–22	Non-quantifiable	Orphan drug
5th generation cephalosporin + b lactamase inhibitor	J01DI54	ceftolozane tazobactam	cIAI, AP, cUTI	Adult	Phase III	Active	NI	jul.–16	*	I	S	V	+		jun.–16	LR	nov.–22	Considered proven	Reserve ATB
5th generation cephalosporin + b lactamase inhibitor	J01DI54	ceftolozane tazobactam	cIAI, AP, cUTI, HAP, VAP	Adult	Phase III	Active	NI	jan.–20	*	R	S	III	+	LR	dec.–19	LR			
diaminopyrimidines	J01EA01	trimethoprim	uUTI	Female, adolescent, adult	Biblio			jun.–16	*	I	S	V	−						
glycylcyclines	J01AA12	tigecycline	cIAI, ABSSSI	Child (8–18 yrs)	Phase II	SA	PK	jul.–16	*	E	S	V	−						
3rd generation cephalosporin + b lactamase inhibitor	J01DD52	ceftazidime avibactam	cIAI, cUTI, AP, HAP, VAP, R-GNB	Adult	Phase III	Placebo	NI	nov.–16	*	I	S	IV	+		aug.–22	LR	nov.–22	Considered proven	Reserve ATB
3rd generation cephalosporin + b lactamase inhibitor	J01DD52	ceftazidime avibactam	cIAI, cUTI, AP, HAP, VAP, R-GNB	Adult	Phase III	Placebo	NI	janv.–20	*	R	S	III	+	LR					
glycopeptide	J01XA04	dalbavancin	ABSSSI	Adult	Phase III	Active	NI	dec.–16	*	I	S	V	−				feb.–24	Considered proven	Reserve ATB
5th generation cephalosporin	J01DI02	ceftaroline	ABSSSI	Child (2 mo–18 yrs)	Phase III	Active	NI	apr.–17	*	E	S	IV	−						
5th generation cephalosporin	J01DI02	ceftaroline	CAP	Child (2 mo–18 yrs)	Biblio			apr.–17	*	E	NS		−						
5th generation cephalosporin	J01DI02	ceftaroline	ABSSSI	Infant (birth–2 mo)	Phase II	SA	PK	sept.–20	*	E	S	IV	−						
b-lactams broad spectrum	J01CA08	pivmecillinam	uUTI	Female adult	Biblio			oct.–17	*	R	S	V	−						
Carbapenem + b lactamase inhibitor	J01DH52	meropenem vaborbactam	cIAI, cUTI, HAP, VAP, R-GNB	Adult	Phase III	Active	NI	jan.–20	*	I	S	III	+		nov.–19	MDR-aerobic GNB			
antimycobacterial	J04AK05	bedaquiline	MDR-TB	Adolescent (12–18 yrs)	Phase II	SA	PK	sept.–20	*	E	S	III	+	LR			sep.–21	Non-quantifiable	Orphan drug
carbapenem + b lactamase inhibitor	J01DH56	imipenem cilastatin relebactam	R-GNB	Adult	Phase III	Active	NI	sept.–20	*	I	S	III	+	LR	oct.–20	LR	nov.–22	Considered proven	Reserve ATB
fluoroquinolone	J01MA23	delafloxacin	ABSSSI	Adult	Phase III	Active	NI	dec.–20	*	I	S	V	+	LR	jan.–21	LR			

*Note*: *: hyperlink to the French HTA opinion; 3GC: Third-generation cephalosporins; ABSSSI: Acute bacterial skin and skin structure infections; AP: Acute pyelonephritis; ATB, antibiotic; CAP: community-acquired pneumonia; CAV: clinical added value; CB, clinical benefit (S substantial, NS no substantial); CAV, clinical added value (CAV III, moderate; CAV IV, minor; CAV V, no therapeutic progress); cIAI: complicated intra-abdominal infection; Comp., comparator (SA, single arm); Context (I, inscription, E, extension: pediatrics; R, reassessment); cUTI: complicated urinary tract infections; ESBLPE: extended spectrum beta-lactamase producing *Enterobacteriaceae*; GNB: Gram-negative bacteria; HAP: Hospital-acquired pneumonia; INN: International nonproprietary name; MA: Marketing Authorisation; MDR-TB: Multidrug-resistant tuberculosis; NI: noninferiority; Obj., objective (S, superiority, NI, noninferiority, PK, pharmacokinetics); PHI, Public Health Impact (+, yes, − no); Population (Yrs, years, mo, months); TC-HAS: Transparency Committee of the French National Authority for Health; uUTI: Uncomplicated urinary tract infections; VAP: ventilator-associated pneumonia.

The reassessment of a drug is done at the request of manufacturer or by self-entry of the TC-HAS. Following an initial assessment in a given indication, new clinical data are provided by the laboratory so that the TC-HAS can review its previous conclusions (position in the therapeutic strategy, CB, PHI, CAB, or target population).

Proven or strongly suspected methicillin resistance refers to situations where a bacterial strain is known to be resistant to methicillin or is strongly suspected to possess such resistance. Proven resistance: Indicates that laboratory testing or other diagnostic methods have confirmed the presence of resistance in the bacterial strain. Suspected resistance: Implies that there is a high level of suspicion based on clinical or epidemiological factors (i.e. history of previous infections, healthcare exposure, proximity to outbreaks, the presence of invasive devices, close contact with infected individuals, chronic illnesses, recent antibiotic use, prolonged hospital stay, a history of MRSA in the community, and clinical severity), even if definitive laboratory confirmation may not be available.

TC-HAS deemed that the CB was considered substantial for all but one application. A positive reimbursement decision was reached for some or all MA indications: 8 for novel therapeutic agents, 1 for reassessment based on new data, 5 for additional indications for previously approved drugs, and 1 for reassessment and supplemental indication. Unfavorable opinion for reimbursement was attributed once in 2017 to ceftaroline in community-acquired pneumonia due to the lack of data on efficacy in community-acquired methicillin-resistant *Staphylococcus aureus* (MRSA) pneumonia or against strains of *Streptococcus pneumoniae* not susceptible to penicillin, and to the existence of therapeutic alternatives with a narrower spectrum.

**Figure 1.**
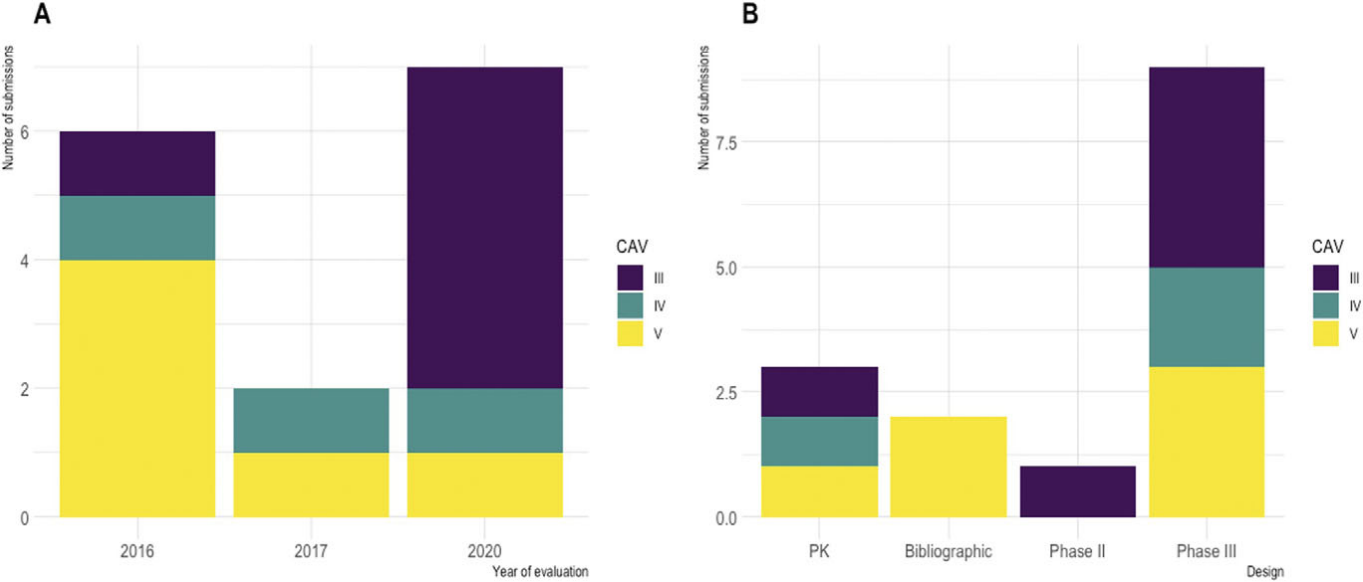
Appraisal by TC-HAS in terms of clinical added benefit of the antibiotic assessed according to date (A) or study design (B). Part A of Figure 1 shows that in 2020, there was more antibiotic with significative clinical added value (CAV III or IV) than in previous years. Part B of Figure 1 shows that the recognition of innovation, resulting in a significative CAV, was supported by more advanced clinical developments, in particular with the completion of phase III clinical trials. The clinical added value (CAV) is an assessment of the therapeutic (or diagnostic) progress provided by a medicinal product – notably in terms of efficacy or safety – compared with existing alternatives. It measures the medical added value of the medicine compared with existing therapies: this assessment is a snapshot at a given point in time within an environment that may evolve. It may be rated major (CAV level I), substantial (CAV level II), moderate (CAV level III), minor (CAV level IV), or no improvement (CAV level V), with the latter level corresponding to no therapeutic progress. The clinical benefit (CB) of a medicinal product in a given indication is assessed on the basis five factors: the efficacy and adverse effects of the medicinal product; its place in the therapeutic strategy, particularly with respect to the other therapies available; the seriousness of the disease targeted by the medicinal product; the preventive, curative or symptomatic nature of the medicinal product; the public health benefit of the medicinal product.

The CAV was set as moderate (level III) in 6 (40 percent) assessments, minor (level IV) in 3 (20 percent), and absent (level V) in 6 (40 percent), increasing over the year of assessment: moderate CAV increased from 16.7 percent of assessments in 2017 to 71.4 percent in 2020 (Figure 1A). Such moderate CAVs were supported by more advanced clinical developments, as illustrated by phase III clinical trials (Figure 1B). Among the valued antibiotics, and among the 8 with PHI, TC-HAS restricted the prescription of five as reserve antibiotics, that is, restricting their use where there is a major unmet medical need (Table 3). These new antibiotics were either active against MDR carbapenemase-producing enterobacterales (CPE) or resistant to at least one carbapenem. However, no major or important CAV was granted due to significant methodological flaws in clinical trials. Actually, regardless of the clinical trial evidence, a positive reimbursement decision was more likely if the drug addressed an unmet medical need as defined by the severity of the disease, and the availability of alternative treatments, two points that specifically apply to the treatment of MDR infections with antibiotics (14).

### Comparison between G-BA, NICE, and TC-HAS

The qualitative comparison of TC-HAS appraisals with those from the G-BA (47–55) and NICE (56–63) only concerned the 8 antibiotics with available assessments, namely 4 beta-lactams combined with a beta-lactamase inhibitor, 1 glycopeptide, 2 antituberculosis drugs, and 1 fluoroquinolone (Table 3).

For the latest generations of antibiotics targeting MDR bacteria (avibactam/ceftazidime, ceftolozane/tazobactam, imipenem/cilastatin/relebactam, and – only for NICE, meropenem/vaborbactam-), favorable reimbursement opinions were close whichever the country. NICE restricted the imipenem/cilastatin/relebactam combination for Gram-negative aerobic infections in adults, and the ceftolozane/tazobactam combination for hospital-acquired pneumonia (HAP), cIAI, and acute pyelonephritis when treatment options are limited. The TC-HAS, G-BA, and NICE offered reimbursement of avibactam/ceftazidime, ceftolozane/tazobactam, and imipenem/cilastatin/relebactam only as reserve antibiotics to limit the emergence of resistance.

Otherwise, there were discrepancies across the countries. Dalbavancin was reimbursed by the G-BA for the treatment of ABSSSI in adults and children over 3 months of age as a reserve, while only for adults with proven or suspected severe infections, *Staphylococcus* pathogen or methicillin resistance by the TC-HAS (no opinion from NICE). Delamanide and bedaquiline were reimbursed by TC-HAS and G-BA for all MA indications, except bedaquiline in children aged 5 to 12 years for which the TC-HAS did not receive any demand (no opinion from NICE). Both delamanide and bedaquiline, the CAV was nonquantifiable, according to the G-BA assessment procedure for orphan drugs (Table 3). Last, delafloxacin was reimbursed in all MA indications by NICE but only in adult ABSSSI by the TC-HAS, but restricted to reserve antibiotic by both the NICE and TC-HAS, with the recommendation to use this new fluoroquinolone when other conventional treatments are not suitable (no opinion from G-BA).

## Discussion

In this study, we analyzed the appraisals of new antibiotics assessed by the French TC-HAS from 2016 to 2020, then compared these appraisals to those from the NICE and the G-BA. Overall, the TC-HAS issued 14 positive opinions on 12 different antibiotics for reimbursement by national solidarity, with only one decline due to a lack of clinical data (ceftaroline, CAP). For 60 percent (9/15) of the evaluated antibiotics, the TC-HAS graded the CAV as moderate or minor from robust preclinical and clinical data, making them available to patients. Nevertheless, the scope of reimbursement was restricted as a reserve for some antibiotics to guide their use by healthcare professionals and patients and to prevent the development of MDR bacteria by documented prescription on antibiograms in accordance with AMR treatment guidelines (64).

A qualitative comparison of TC-HAS opinions with those of NICE and G-BA was possible for only 8 of the 12 antibiotics, thus avoiding any quantitative valid comparison, given different assessment rules and public availability across countries. Notably, G-BA exempted 4 reserve antibiotics from the whole HTA process (ceftazidime/avibactam, ceftolozane/tazobactam, dalbavancin, and imipenem/ cilastatin/ relebactam). Despite the differences in the assessment methods across the different countries, the analysis shows an equivalent positioning of antibiotics, for instance, restricting the use of the latest molecules targeting CPE for pathogens resistant to other antibiotics similarly by the 3 HTA bodies.

Global efforts led by the WHO and European HTA bodies are underway to assess antibiotics clinical value and financing, considering the impact of AMR. HTA bodies should advice healthcare professionals on how to use new antibiotics to preserve them from the risk of the emergence of resistance. In this setting, this paper is an original work, given the very few publications addressing this issue. (65) Our analyses should enhance the dialog of HTA bodies and manufacturers regarding antibiotics directed toward MDR bacteria that may improve clinical evidence further provided in future demands. There are indeed still expectations on the research and development side because the market launch of new antibiotics remains marginal compared to therapeutic areas such as oncology. The updated TC-HAS guidance appears to be one of the answers to support manufacturers in the development of antibiotics targeting MDR bacteria.

During the period of analysis (2016–2020), no new class of antibiotic was developed and marketed by the manufacturer. The clinical development of the latest antibiotics has mainly focused on cIAI and cUTI. This is likely explained by the facility to reach these target organs, compared to the lung or bone tissue, for instance. Although the conduct of a clinical trial evaluating an antibiotic targeting a specific pathogen requires an appropriate methodology, with consideration of local antibiogram data, it likely provides an improved level of evidence of the added therapeutic value of a new antibiotic targeting MDR bacteria. Since this work, only one new antibiotic targeting MDR bacteria has been made available (céfidérocol, 2021).

This comparative analysis showed a roughly similar approach between TC-HAS, G-BA, and NICE in managing the risk of the emergence of AMR. The TC-HAS has assessed the new antibiotics targeting MDR bacteria by granting them a PHI and a moderate CAV. The G-BA has exempted these antibiotics from a full HTA procedure with a reserve antibiotic status. NICE has proposed these antibiotics for reimbursement as a last resort only in the absence of suitable alternative treatment options. Aside from the valuation or absence of a full HTA procedure, the three HTA bodies positioned these antibiotics as a last resort when therapeutic options are limited to avoid massive use of these antibiotics and the risk of emerging resistance. This is in agreement with WHO, which emphasized the importance of developing new antibiotics to combat resistant strains while advocating the prudent use of existing antibiotics to minimize resistance emergence. This is also consistent with the recommendations of the Infectious Diseases Society of America, particularly for antibiotics targeting carbapenemase-producing bacteria (66). However, it cannot be universal due to the heterogeneity in the epidemiology of MDR bacteria. In European MAs, a statement “Consideration should be given to official guidance on the appropriate use of antibacterial agents” has been added to section 4.1 “Therapeutic indications of the summary of product characteristics” to give the EU member states the opportunity to define the place of the antibiotic in the therapeutic strategy according to the alternatives available and local ecology. For example, CPE, a critical priority bacteria according to the WHO, has a heterogeneous spread between countries (primary and secondary epidemiological focus) and even between regions or sites (67,68). Thus, given the specificity of microbial ecology at a local, regional, or national level, the meeting medical need and therefore the reimbursement decision may differ from one country to another.

One of the limitations of this work was the analysis period between 2016 and 2020, with only 12 different antibiotics evaluated by the TC-HAS. It also highlights the low number of developed new antibiotics compared for instance to the high number of drugs dedicated to oncology. The comparison of decisions of three HTA bodies that use different approaches to drug assessment, with different times of assessment, could be another limitation. Given that HTA opinions are public, it is also possible that a decision on a drug by a HTA body is influenced by the position of the other HTA bodies. Nevertheless, this study could serve as a pivotal element in steering research and development efforts toward producing solutions that effectively address priority public health concerns in combating antibiotic resistance.

## Perspectives and conclusion

In conclusion, each of the French, German, and UK HTA bodies has adopted its own strategy for the assessment of antibiotics. In France, the TC-HAS has developed a specific HTA guidance to highlight for the firms the determinants of the clinical value of antibiotics as the indirect way to promote the development of antibiotics targeting unmet and priority needs. In Germany, the G-BA exempts antibiotics from full HTA procedure as long as they meet the criteria for reserve antibiotics and according to the health priorities defined by the RKI. England places emphasis on the economic dimension of making the new antibiotics available, with both the NHS and NICE proposing a financing model whereby manufacturers developing new antibiotics are paid a fixed annual fee, irrespective of the actual sales volume of the antibiotic. On a European scale, the EU-JAMRAI is discussing innovative financing pull-incentives mechanisms to encourage manufacturers to reinvest in research and development on new antibiotics. These mechanisms must also address the financial sustainability of governments and their healthcare systems. Facing the global AMR burden, countries should implement action plans to prevent and control antibiotic resistance.

This paper contributes to health policymaking in Europe regarding new antibiotic assessments, to the criteria considered by HTA decision-makers in France, England, and Germany. The consistent positions on antibiotic assessment adopted by TC-HAS, G-BA, and NICE are encouraging in view of the application of the new European HTA regulation (Regulation (EU) 2021/2282 of December, 15, 2021), even though NICE is not part of this joint European HTA organization (69). These similar approaches should favor the development of new products and innovative technologies that meet public health needs and contribute to the fight against AMR. Other incentive mechanisms to stimulate research and development of new antibiotics are being explored, including financial ones such as the USA, considering pricing new antibiotics independently of prescription volume in the form of an innovative payment contract with Pasteur Act (70).

## Supporting information

Dumont et al. supplementary material Dumont et al. supplementary material

## Supplementary material

The supplementary material for this article can be found at http://doi.org/10.1017/S0266462324000552.
