# Supplementary material for: How do we respond to the threat of multidrug-resistant bacteria? Comparison of antibiotic appraisals from 2016 to 2020 of the French, English, and German HTA agencies
Source: Int J Technol Assess Health Care. 2024 Dec 9;40(1):e72. doi: 10.1017/S0266462324000552 (PMC11703620; doi:10.1017/S0266462324000552)
Supplement: Dumont et al. supplementary material [file S0266462324000552sup001.docx]

**Supplementary materials Table S1: WHO list of multi-resistant pathogens**

| PRIORITY | PATHOGENS | RESISTANCE |
| --- | --- | --- |
| 1. Critical | *Acinetobacter baumannii* | Carbapenem |
|  | *Pseudomonas aeruginosa* | Carbapenem |
|  | *Enterobacteriaceae** | Carbapenem, 3rd generation cephalosporins |
| 1. High | *Enterococcus faecium* | Vancomycin |
|  | *Staphylococcus aureus* | Methicillin, vancomycin |
|  | *Helicobacter pylori* | Clarithromycin |
|  | *Campylobaccter spp.* | Fluoroquinolones |
|  | *Salmonella spp.* | Fluoroquinolones |
|  | *Neisseria gonorrhoeae* | 3rd generation cephalosporins, fluoroquinolones |
| 1. Moderate | *Streptococcus pneumoniae* | Penicillin |
|  | *Haemophilus influenzae* | Ampicillin |
|  | *Shigella spp.* | Fluoroquinolones |

*Including *Citrobacter spp*., *Enterobacter spp*., *Escherichia coli*, *Klebsiella spp*., *Proteus spp*., *Providencia spp*., *Morganella spp*. et *Serratia spp*.

**Supplementary materials Table S2: NON-EXHAUSTIVE LIST OF MULTIDRUG-RESISTANT BACTERIAL PATHOGENS (2021) - ADAPTED FOR GERMANY ACCORDING TO THE WHO PRIORITY PATHOGEN LIST**

*Acinetobacter baumannii, CR*

*Burkholderia cepacia complex*

*Campylobacter spp, FQR*

*Citrobacter spp, 3GCR*

*Enterobacter spp, 3GCR*

*Enterobacter spp, CR*

*Enterococcus faecium, VR*

*Escherichia coli, 3GCR*

*Escherichia coli, CR*

*Haemophilus influenzae, AmpR*

*Helicobacter pylori, ClaR*

*Klebsiella spp, 3GCR*

*Klebsiella spp, CR*

*Morganella spp, 3GCR*

*Neisseria gonorrhoeae, 3GCR*

*Neisseria gonorrhoeae, FQR*

*Proteus spp, 3GCR*

*Providencia spp, 3GCR*

*Pseudomonas aeruginosa, CR*

*Salmonella Typhi, FQR*

*Serratia spp, 3GCR*

*Shigella spp, FQR*

*Staphylococcus aureus, MR*

*Stenotrophomonas maltophilia*

*Streptococcus pneumoniae, PR*

3GC = third-generation cephalosporin-resistant; AmpR = ampicillin-resistant; ClaR = clarithromycin-resistant; CR = carbapenem-resistant; FQR = fluoroquinolone-resistant; MR = methicillin-resistant; PR = penicillin-resistant; VR = vancomycin-resistant
